# Why is next-generation sequencing essential in modern virology?

**DOI:** 10.1099/jgv.0.002050

**Published:** 2024-11-21

**Authors:** Charlotte Lefèvre, Nerea Irigoyen

**Affiliations:** 1 Division of Virology, Department of Pathology, University of Cambridge, Tennis Court Road, Cambridge, UK

**Keywords:** next-generation sequencing, ribosome profiling, RNA viruses, RNASeq, viral contamination

## Abstract

A contaminated viral stock results in considerable loss of time, resources and financial means and is generally discovered only by chance after years of research. Thus, it is necessary to implement a technique that can detect contamination without prior knowledge or assumptions, such as next-generation sequencing (NGS). Here, we describe the discovery of a cross-contaminated viral stock from a biological repository of an African Zika virus isolate with Toscana virus after performing NGS on infected cells. In addition, we also describe the consequences that we faced using this contaminated stock. These included the economic and time loss to the lab that needed to repeat all previously performed experiments, the generation of biologically flawed results with a subsequent potential retraction and the severe risk of infection of lab members who manipulated the contaminated stock.

## Data Availability

Library amplicons were constructed using a small RNA cloning strategy adapted for Illumina smallRNA v2 to allow multiplexing [1]. Amplicon libraries were deep-sequenced using an Illumina NextSeq500 platform. Ribo-Seq and RNA-Seq data have been deposited in the ArrayExpress database (http://www.ebi.ac.uk/arrayexpress): E-MTAB-14055 and E-MTAB-14056.

## Introduction

Biological stocks from public repository archives are assumed to be clean and contaminant-free. However, this may not always be the case, and unknown contamination of biological samples can have significant consequences.

Non-suspected contaminants will have biological effects on the system that are aimed to be studied, and the consequent flawed results will be interpreted, published and shared with the scientific community, leading to novel ideas and more extensive studies based on these data, which can have a substantial impact down the line. Moreover, contamination by certain viruses and bacteria can breach the established biosafety levels and pose severe risks for scientists manipulating the contaminated samples. Amongst the most critical elements to be contaminated in a biological laboratory are biological stocks, and a contaminated stock is normally discovered by coincidence after years of research, resulting in a significant number of flawed conclusions and a considerable loss of time, resources and financial means. Therefore, an early detection of unknown contamination is crucial.

Before the surge of next-generation sequencing (NGS), high-throughput sequencing or deep sequencing, only known or suspected contaminants could be detected in biological samples. For example, inferred fungal, bacterial or viral contamination was efficiently detected with high specificity and sensitivity using *in vitro* assays (e.g. PCR) or electron microscopy (e.g. based on morphology) [2,4]. However, accurate detection of a whole range of contaminants might be challenging by using only these techniques [3], as the list of PCR primers for detecting contaminants is too short, as it includes only known or suspected contaminants, resulting in limited detection (i.e. in our case, the list of PCR primers are limited to *Escherichia coli*, mycoplasma and other viruses investigated in the lab such as MHV-A59). The use of electron microscopy for detecting and analysing virus morphology within a sample lacks sensitivity and depends on the operator’s expertise. Therefore, a technique encompassing a broader detection range without prior knowledge or assumptions about contaminating species must be implemented.

NGS technology amplifies and sequences fragmented nucleic acid reads, making it a highly sensitive method for discovering, detecting and identifying pathogens of both known and unknown origin or species [5]. Generally, ~70–90% of the sequenced reads will map to reference genomes of known species, leaving ~10–30% as ‘unmapped’ reads [6]. These ‘unmapped’ reads will not only be attributed to (non-)suspected contaminants but might also contribute to the discovery of novel species, such as viral species that share sequence homology with known viruses [7,8].

## Detection of contaminants in the lab

Samples from collaborators or biological archives are routinely tested upon arrival in our lab to assess their purity and quality. For example, cell culture batches are tested for mycoplasma contamination, and viral stocks are verified by viral genome expression, Western blotting, plaque assays and Sanger sequencing. However, these tests cannot detect unknown or (non-)suspected contaminants.

**Fig. 1.**
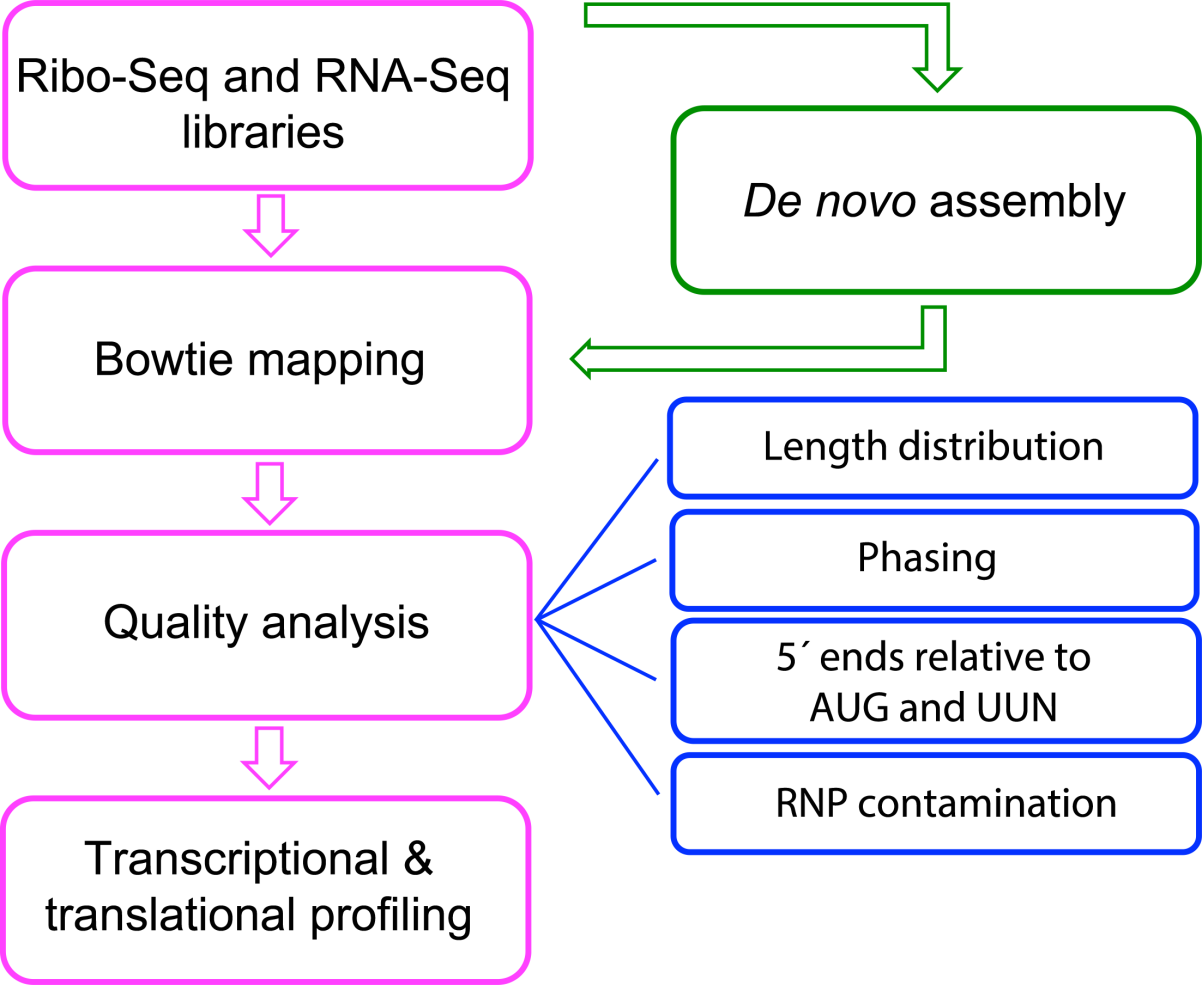
Bioinformatic pipeline for Ribo-Seq and RNA-Seq data processing. RNP: ribonucleoprotein.

One of the most significant advantages of our lab is the possibility to generate NGS data from samples received from collaborators or biological archives that are subjected to a rigorous quality control analysis. In our NGS pipeline (Fig. 1) [1], the first analysis includes a Bowtie mapping of reads to reference genomes that verifies read count distribution across species of known origin, whereas the remaining reads are mapped to a ‘contaminants’ database of suspected origin. The latter database contains sequences of common contaminants (e.g. mycoplasma) and sequences of microorganisms that are commonly used in the lab (e.g. *E. coli*). However, if a substantial number of reads remain in the ‘unmapped’ fraction, this will be considered contamination of unknown origin and reads will be subjected to a *de novo* assembly [i.e. assembled contiguous sequences (contigs)] using the bioinformatic tool Trinity that will be mapped using Basic Local Alignment Search Tool (blast) [9]. If a high level of similarity is found between the contigs and a specific genome/sequence of a particular species or microorganism, this will likely indicate the origin of the ‘unmapped’ reads and the potential ‘contaminant’ in that specific sample. Then, Bowtie mapping will be carried out using the identified genome/sequence of the contaminant to determine the number of reads originating from this source. Once this new contaminant is identified, it will be added to the ‘contaminants’ database for quality control analyses of future datasets.

By applying these analyses, the nature and level of contamination can be determined. This can indicate the extent to which the contaminants can influence the obtained results. For example, a maximum of 1% of reads mapping to mycoplasma sequences, which is typical for lab-generated datasets, is accepted, as these sequences will partly include reads that are not only mycoplasma-specific but also highly conserved bacterial sequences [1]. However, results obtained from datasets containing higher levels of mycoplasma contamination should be carefully interpreted, as mycoplasma infection influences host cell biology by depriving cells of nutrients and altering the expression of cellular genes [10].

In this article, we provide an example of how the implementation of NGS techniques has identified contamination of a viral stock provided by a biological repository with another virus and its critical consequences.

## Contamination of an African Zika virus isolate with Toscana virus

**Fig. 2.**
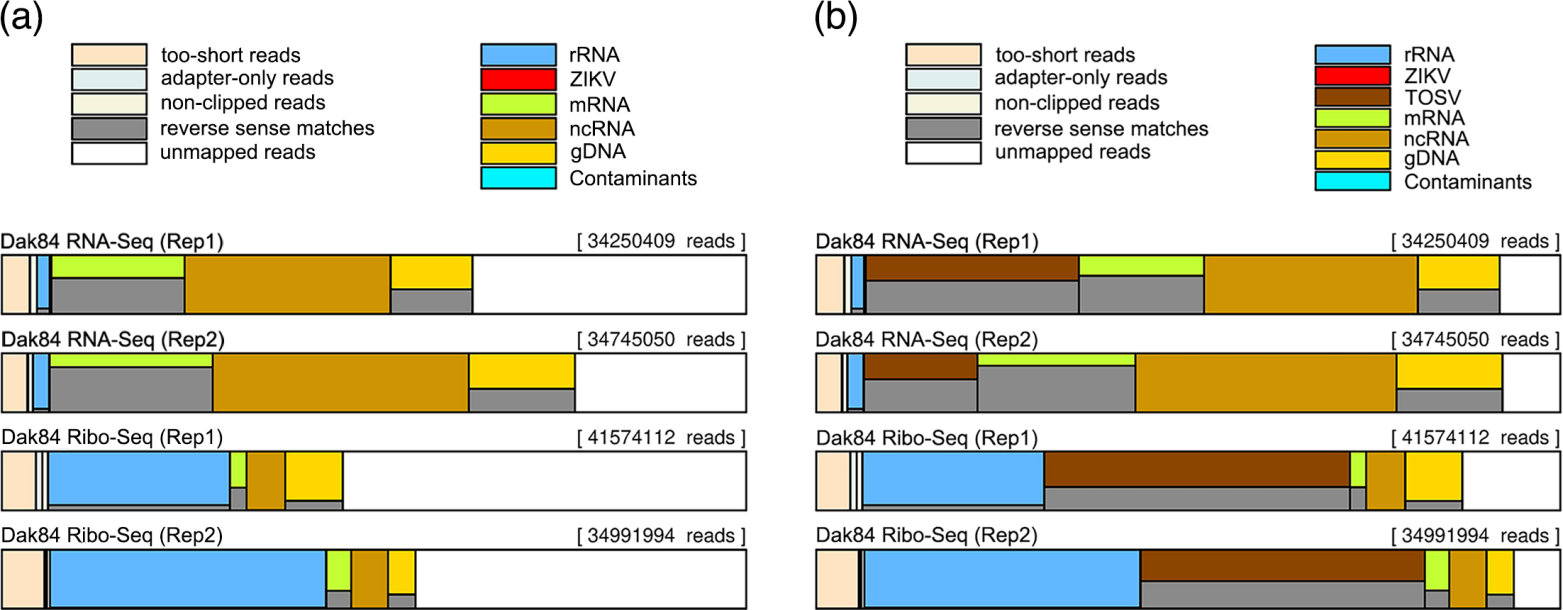
Library composition plots of Ribo-Seq and RNA-Seq libraries obtained from Dak84-infected Vero cells. (**a**) Proportion of discarded reads and reads mapping to the rRNA, ZIKV RNA, mRNA, ncRNA, gDNA and contaminants databases using Bowtie version 1.2.3. (**b**) Proportion of discarded reads and reads mapping to rRNA, ZIKV RNA, TOSV RNA, mRNA, ncRNA, gDNA and contaminants databases. Rep1 and Rep2 indicate replicate 1 and replicate 2, respectively.

Upon receipt in the lab from the EVAg repository, the African Zika virus (ZIKV) isolate Dak84 (001 V-02028) was assessed by plaque assay, viral RNA analysis and Western blotting, which confirmed its identity. Then, this viral stock was used to infect Vero cells to generate Ribo-Seq and RNA-Seq datasets. Bowtie mapping of these libraries resulted in a high proportion of reads in the ‘unmapped’ fraction, ranging from 47.59 to 57.79% and 24.02 to 39.59% for Ribo-Seq and RNA-Seq datasets, respectively (Fig. 2a). After the *de novo* assembly, eight contigs were found using these ‘unmapped’ reads, which mapped – at the nucleotide level using blast – to three different accession numbers: KU204981, KU204892 and KU204893 corresponding to the three genome segments of the Toscana virus strain Nice/113 (TOSV), a negative sense single-stranded segmented *Phlebovirus* [11].

Then, these datasets were re-mapped to the previously mentioned databases, including the genome sequences of TOSV. As observed in Fig. 2b, the library composition shows that 40.82–43.79% and 15.91–30.05% of reads mapped to the TOSV genome (dark brown) in Ribo-Seq and RNA-Seq libraries, respectively, and only 14% of reads failed to map to any of the databases.

As ZIKV and TOSV are both arboviruses that share the same mosquito vector, we hypothesize that the *Aedes taylori* mosquito used to isolate Dak84 may have also been co-infected with TOSV. However, we cannot rule out that co-contamination was acquired during the serial passage of the viral isolate in several cell lines [i.e. once in *Aedes pseudoscutellaris* cells (AP61), once in C6/36 cells and thrice in Vero cells before the distribution of this virus stock] [12].

## Consequences of using a TOSV-contaminated African ZIKV Dak84 stock

Using a viral stock contaminated with an unknown source can have multiple consequences, ranging from an economic loss to the lab, as it will need to repeat those experiments to a severe risk of infection to lab members manipulating that stock. Here, we describe some consequences that we faced:

### Consequence 1: loss of time and economic resources

In the contaminated samples, more than a quarter of the Ribo-Seq and RNA-Seq reads were assigned to the TOSV genome (Table 1). Since NGS is a quantitative method, the prevalence of a species in a sample is proportionate to the number of deep-sequenced reads originating from that species. This suggests that the number of TOSV particles in the Dak84 stock sample was substantially higher than that of ZIKV particles or that TOSV could infect the Vero cells more efficiently, outcompeting ZIKV replication. Consequently, the number of reads mapping to the ZIKV genome was low, ranging from 0.03 to 0.25% of the total reads, leading to very low genome coverage, especially in the Ribo-Seq samples. This rendered these samples unusable for the analysis of the ZIKV translatome.

**Table 1.** Percentage of reads of two independent (replicates 1 and 2, Rep 1 and Rep2) ribosome profiling (Ribo-Seq) experiments coupled with RNA-Seq mapping to different databases. Percentage of reads mapping to host rRNA, mRNA, ncRNA, gDNA, ZIKV RNA, TOSV RNA and contaminants databases. It also includes reads that failed to align. Alignment was done using Bowtie version 1.2.3

	Ribo-Seq (Rep 1)	Ribo-Seq (Rep 2)	RNA-Seq (Rep 1)	RNA-Seq (Rep 2)
**rRNA (%**)	26.03	39.64	1.77	2.27
**ZIKV (%**)	0.03	0.06	0.25	0.08
**TOSV (%**)	43.79	40.82	30.05	15.91
**mRNA (%**)	2.34	3.52	17.66	22.15
**ncRNA (%**)	5.58	5.35	30.17	36.61
**gDNA (%**)	8.20	3.92	11.52	14.85
**Contaminants (%**)	0.03	0.01	0.01	0.00
**Unmapped (%**)	14.01	6.68	8.58	8.13

Importantly, TOSV genome segments are entirely covered by nucleoproteins (NPs) to form ribonucleoprotein (RNP) complexes associated with viral RNA. It has been reported that the TOSV nucleocapsid protein (N) can also bind single-stranded RNA with nanomolar affinity without sequence specificity [13]. Therefore, TOSV NPs could associate with ZIKV or cellular mRNAs to form RNPs [14,15]. These RNPs, with a similar molecular weight as ribosome–mRNA complexes, co-sediment with monosomes during the ultracentrifugation step of Ribo-Seq library preparation, which in turn leads to a large proportion of mRNA reads associated with contaminating TOSV NPs. Thus, no conclusions could be formulated on the African ZIKV translational profile. Additionally, it should not be excluded that TOSV might influence ZIKV gene expression. Therefore, the combination of low ZIKV reads and high RNP contamination meant that these datasets were unsuitable for analysing the ZIKV transcriptome and translatome, resulting in an economic loss and time loss for the lab.

### Consequence 2: generation of biologically flawed results

RNA-activated protein kinase (PKR) is a kinase triggered by dsRNA, such as viral dsRNA. This dsRNA binds to PKR, which dimerizes and leads to autophosphorylation, forming phospho (p)-PKR [16]. p-PKR subsequently causes the phosphorylation of the eukaryotic initiation factor 2α (eIF2α), leading to the inhibition of cellular protein translation [17].

The ZIKV dsRNA intermediate, synthesized during genome replication, can strongly trigger PKR activation, leading to eIF2α phosphorylation and translational shut-off, as reported for the American ZIKV lineage [18,20]. Thus, in the lab, we wanted to verify whether the African isolate Dak84 would also induce PKR activation.

**Fig. 3.**
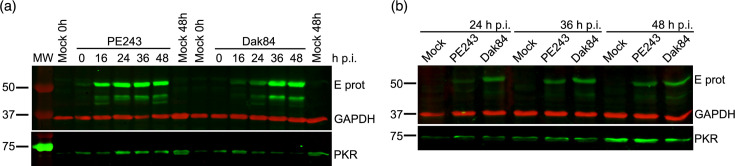
Analysis of PKR degradation by ZIKV. Western blot analysis of ZIKV E protein (anti-E, GeneTex, GTX133314, 1 : 1000) and PKR (anti-PKR, Abcam, ab184257, 1 : 1000) of Vero cells infected with PE243 (American ZIKV isolate) and Dak84 (African ZIKV isolate) at MOI 3 and harvested at indicated time points. GAPDH (anti-GAPDH, Sigma-Aldrich, G8795, 1 : 20 000) was used as a loading control. In **a**, Dak84 isolate is derived from the biological repository (TOSV contaminated), whereas in **b**, Dak84 isolate was plaque-purified twice to remove TOSV contamination and its purity was verified by RT-qPCR and NGS.

Vero cells were infected with the American ZIKV isolate, PE243 and the TOSV-contaminated African isolate, Dak84, at a multiplicity of infection (MOI) 3 and harvested at different time points. As observed in Fig. 3a, PKR is detected in cells infected with both viruses. However, at 24 h post-infection (h p.i.), PKR levels in TOSV-contaminated Dak84-infected cells decrease compared to those in PE243-infected cells. This suggests that the African ZIKV could counteract PKR activation, probably via protein degradation, potentially affecting innate immune responses.

However, this conclusion was revisited after discovering the TOSV contamination in the Dak84 stock. A purified stock for Dak84 was used to infect fresh Vero cells alongside PE243 during a time course to test this. Fig. 3b shows that PKR was no longer degraded in Dak84-infected cells. This indicates that PKR degradation by Dak84 was not ZIKV strain-specific but caused by TOSV infection that forms a complex targeted for proteasomal degradation *via* its non-structural (NS) proteins [21]. Thus, without the discovery of TOSV contamination, we might have mistakenly suggested that African ZIKV isolates could degrade PKR, generating a biological flaw that would have needed to be retracted in the future.

### Consequence 3: risk of infection

In most cases, TOSV infection produces flu-like symptoms, including fever, photophobia, headache, red eyes and stiff neck. However, it can also infect the central nervous system in some cases, causing meningitis and meningoencephalitis. This poses a severe risk to the health of lab members who are exposed to a dangerous pathogen without the required prophylactic measures. In this case, ZIKV and TOSV are classified as containment level 2 pathogens, so the risk of exposure was already contained. However, this might not always be the case with unknown contaminants.

## Conclusion

This work underscores the necessity of subjecting biological samples to deep sequencing, particularly as it becomes more accessible and affordable.

NGS can be overwhelming in principle, as researchers need proper training in library preparation and bioinformatic analysis. However, these inconveniences can be rapidly overcome using different sequencing facilities at public research institutions or private companies that will help in library preparation and downstream analysis. For example, total RNA library preparation, a NextSeq 2000 run and bioinformatic analysis for 24 samples will cost £8843 (£368.45 per sample) with a turnaround time of ~7 days at Cambridge University sequencing facility, Cambridge Genomic Services (October 2024 quotation). This price can even be reduced to ~£150 when using private companies such as BGI Genomics. The price includes library preparation, sequencing and bioinformatic analysis with a turnaround time of ~25 days (October 2024 quotation).

The power, versatility and sensitivity of NGS in identifying the nature and level of contamination in biological samples make it an indispensable tool for detecting known, suspected and unknown contaminants. Hereby, consequences of contamination of stock samples, such as the publication of flawed results, their irreproducibility and potential retraction and complications with significant impacts, such as time and economic losses or breaching biosafety levels, can be easily avoided.

We would like to indicate that we have contacted EVAg about the elimination of this sample from its catalogue, and it is no longer available.

## Abbreviations

blast: Basic Local Alignment Search Tool
MOI: multiplicity of infection
NGS: next-generation sequencing
NPs: nucleoproteins
NS: non-structural
RNP: ribonucleoprotein
TOSV: Toscana virus
ZIKV: Zika virus
